# Identification of Plasma Metabolomic Profiling for Diagnosis of Esophageal Squamous-Cell Carcinoma Using an UPLC/TOF/MS Platform

**DOI:** 10.3390/ijms14058899

**Published:** 2013-04-24

**Authors:** Ran Liu, Yuan Peng, Xiaobo Li, Yi Wang, Enchun Pan, Wei Guo, Yuepu Pu, Lihong Yin

**Affiliations:** 1 Key Laboratory of Environmental Medicine Engineering, Ministry of Education, School of Public Health, Southeast University, Nanjing 210009, Jiangsu, China; E-Mails: pengyuanseu@163.com (Y.P.); xbliseu@163.com (X.L.); yppu@seu.edu.cn (Y.P.); 2 Huaian Center for Disease Control and Prevention, Huaian 223001, Jiangsu, China; E-Mails: wangyihuaian@126.com (Y.W.); enchunpan@163.com (E.P.); 3 The First People’s Hospital of Huaian, Huaian 223000, Jiangsu, China; E-Mail: weiguohy@126.com

**Keywords:** metabolomics, plasma, esophageal squamous-cell carcinoma, UPLC/TOF/MS

## Abstract

Epidemiological studies indicated that esophageal squamous-cell carcinoma (ESCC) is still one of the most common causes of cancer incidence in the world. Searching for valuable markers including circulating endogenous metabolites associated with the risk of esophageal cancer, is extremely important A comparative metabolomics study was performed by using ultraperformance liquid chromatography-electrospray ionization-accurate mass time-of-flight mass spectrometry to analyze 53 pairs of plasma samples from ESCC patients and healthy controls recruited in Huaian, China. The result identified a metabolomic profiling of plasma including 25 upregulated metabolites and five downregulated metabolites, for early diagnosis of ESCC. With a database-based verification protocol, 11 molecules were identified, and six upregulated molecules of interest in ESCC were found to belong to phospholipids as follows: phosphatidylserine, phosphatidic acid, phosphatidyl choline, phosphatidylinositol, phosphatidyl ethanolamine, and sphinganine 1-phosphate. Clinical estimation of metabolic biomarkers through hierarchical cluster analysis in plasma samples from 17 ESCC patients and 29 healthy volunteers indicated that the present metabolite profile could distinguish ESCC patients from healthy individuals. The cluster of aberrant expression of these metabolites in ESCC indicates the critical role of phospholipid metabolism in the oncogenesis of ESCC and suggests its potential ability to assess the risk of ESCC development in addition to currently used risk factors.

## 1. Introduction

Esophageal cancer is among the most common cancer types in the world, ranking eighth in order of occurrence and sixth as the leading cause of cancer mortality [1]. China is one of the countries with high cancer incidence and mortality rate attributed to esophageal cancer. In recent decades, esophageal squamous-cell carcinoma (ESCC), which is the main type of esophageal cancer, has remained a threat in China despite the adoption of nutritional guidelines and water improvement techniques [2]. The etiology of ESCC is still largely unknown, and most ESCC patients have a poor five-year survival rate [3]. Therefore, the identification of novel ESCC biomarkers will be an important strategy in advancing the early diagnosis stage of ESCC and improving the life quality of patients.

Metabolomics focuses on global exploration of endogenous small molecule metabolites as the end products of cellular processes in a biological system, including cell, tissue, organ, or organism [4,5]. Hence, metabolic profiling can illustrate the instantaneous pathological or physiological changes as a supplement to proteomic and transcriptomic profiling for the systematic study of living organisms’ functional status [6–8]. Many studies reveal that the advent of some kinds of cancer is accompanied by metabolic changes, and specific small molecule metabolites profiling can distinguish different clinical and pathological characteristics of cancer such as breast cancer, colon cancer, oral cancer, and prostate cancer [9–15]. Hepatocellular carcinoma and lung cancer could affect liver and lung metabolism and circulating endogenous metabolites [16]. Based on this finding, ESCC may also influence target organ metabolism, and the change in the profile of circulating small metabolites could indicate the risk for ESCC. Therefore, identifying specific circulating small metabolites associated with a risk of ESCC development is a highly valuable step. Few studies have analyzed the effects of ESCC on esophageal metabolism or circulating endogenous metabolites. In the present study, we reported the findings of a comparative metabolomics study performed by using ultraperformance liquid chromatography-electrospray ionization-accurate mass time-of-flight mass spectrometry (UPLC-ESI-TOFMS) to analyze plasma samples from ESCC patients and healthy controls recruited in Huaian.

## 2. Results

### 2.1. Demographic Characteristics

Fifty-three pairs of patients with newly diagnosed, untreated ESCC matched with healthy controls were recruited for the present study. The average age of the patients and controls was 62.6 ± 7.3 and 63.2 ± 8.1 years, respectively. The male to female ratio was 1:4. Of the patients diagnosed with pathological reports, 39 (73.6%) of 53 were diagnosed as well differentiated (I + II) and 14 (26.4%) as poorly differentiated. Lymph node metastases were observed in 17 of the 53 patients (32.1%). ANOVA showed that the average ages of the cancer patients and the controls were not significantly different. Statistical differences were observed between the cases and the controls in the distribution of smoking, alcohol use, and cancer family history. The characteristics of the ESCC patients and the controls are summarized in Table 1.

### 2.2. Plasma Metabolomic Profiles

**Figure 1:**
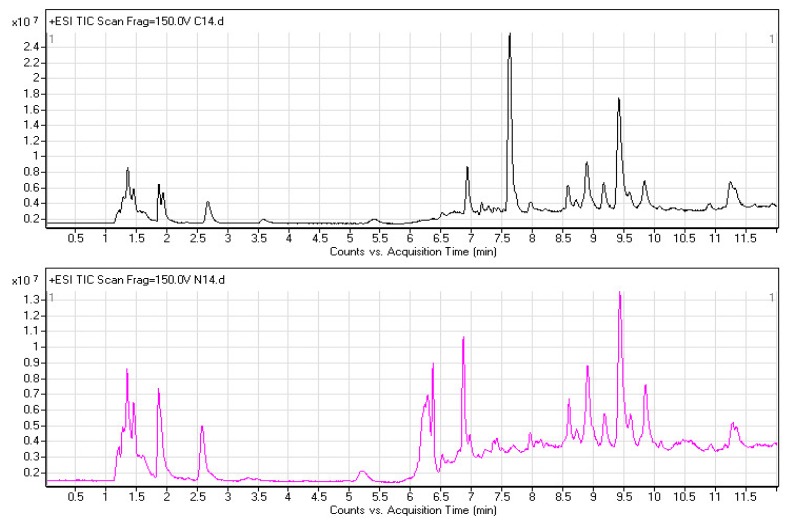
TICs of human plasma samples from an ESCC patient (upper black) and matched healthy control (lower red) in positive ionization mode by using UPLC-ESI-TOF/MS system.

Metabolomics involves studying the processes of all metabolites in healthy or diseased samples, thereby revealing disease-related metabolic pathways. A full-scan detection of plasma metabolites was carried out in the 53 pairs of ESCC patients and healthy controls. Figure 1 shows two typical UPLC-ESI-TOF/MS chromatograms from an ESCC patient (upper) and the matched healthy control (lower). The total ion chromatograms (TICs) exhibited the ideal separation result under the optimized gradient elution procedure and plasma metabolomic profile for each sample, which consisted of approximately 3000 chromatographic peaks (defined by a pair of *m*/*z* value and RT). Significant differences were observed between cancer and control during the retention time (RT) period from 6 to 8.5 min. The features of all chromatographic peaks were extracted for the discovery of metabolic biomarkers associated with ESCC.

### 2.3. Principal Component Analysis Analysis

**Figure 2:**
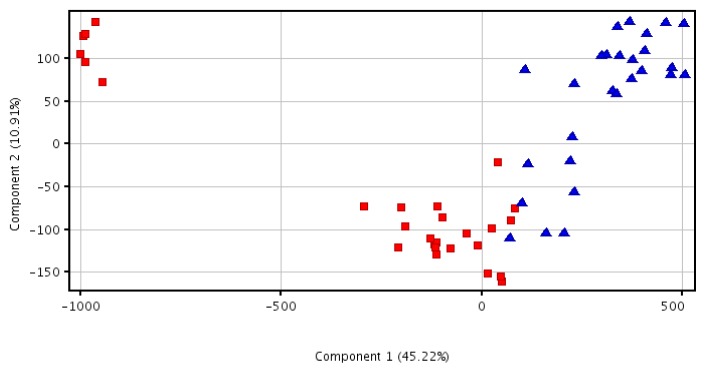
PCA three-dimensional scores plot of plasma metabolic profiling for the top three components which distinguish ESCC patients (blue triangle) from healthy controls (red square).

The acquired metabolomic data were used to perform principal component analysis (PCA), which involves discovering principal components that account for the majority of the differences in the data. As shown in Figure 2, the PCA scores plot showed that data from the samples of different groups tended to cluster and the ESCC group was separated from healthy controls. The first component can account for 45.22% of systematic variance and the second component can account for 10.91% of systematic variance, which exhibited satisfactory performance in a goodness-of-fit test. As shown in the PCA plot of plasma, the healthy controls were clustered into two groups. We verified the characteristics of these two subpopulations and found no differences in parameters such as age, gender, smoking, and drinking history. However, the six samples from a small group were moderately hemolytic, which may interfere with the detection of plasma metabolites. As for the ESCC sample set, several samples (group I) whose PCA scores were close to those of the main healthy controls’ group were separated from the other samples (group II). However, no significant differences were observed among parameters such as age, gender, and poor differentiation between the two subgroups. Lymph node metastases were observed in 33.3% of group II and 25% of group I, which did not indicate statistical significance between the two groups.

### 2.4. Discovery and Identification of Metabolic Biomarkers

Through ANOVA, 39 differentially expressed small molecule metabolites in ESCC patients were distinguished from those of the healthy controls (Table 2, *p* < 0.05); 34 compounds were upregulated and five were downregulated. To control the false discovery rate (FDR) in multiple testing, the Benjamini–Hochberg–Yekutieli procedure was carried out. Thirty significantly differential metabolites were identified with the standard of 0.05; 25 compounds were upregulated and five were downregulated. According to the identity check based on raw data and the features of peaks, the target masses of candidate metabolites identified in the profiling process were searched over a narrow ±10 mDa mass window in the HMDB, METLIN and KEGG databases. The following 15 molecules were identified: phosphatidylserine, 12-oxo-20-dihydroxy-leukotriene B4, 5-β-cyprinol sulfate, L-Urobilinogen, Lithocholic acid taurine conjugate, phosphatidic acid, desmosine (DES)/isodesmosine (IDS), phosphatidyl choline, 9′-carboxy-gama-tocotrienol, Lithocholate 3-*O*-glucuronide, phosphatidylinositol, phosphatidyl ethanolamine, LysoPC(22:2(13Z, 16Z)), Ganglioside GM2(d18: 1/24: 1(15Z)), and Sphinganine 1-phosphate. And 11 molecules were identified with the 5% standard of FDR (Table 2, FDR < 0.05).

Among these compounds, six belong to phospholipids (PLs). PLs are integral parts of the membrane and have important functional, structural, and metabolic roles [17,18]. In this study, all PLs were significantly increased in plasma from esophageal cancer patients unlike in the healthy control. This finding is consistent with results from other cancer studies [19–21]. A high amount of PLs may promote cell membrane anabolism, which accelerates neoplasm cell replication [22] and aberrant cellular lipid composition, and a high quantity may contribute to oncogenesis by altering cellular functions [23,24]. For example, phosphatidylinositol (PIs) and PI derivatives including phosphatidic acid (PA), lysophosphatidic acid (LPA), and phosphatidylinositol phosphate (PIP) are very important molecules to cellular signaling cascades to activate proliferation, maintain survival, and promote migration [25]. Data from the literature showed that the PI profiles of breast cancer cells were significantly different from those of mammary epithelial cells, which suggests that PI molecular species are associated with malignant transformation [26]. Moreover, in cancer cells and solid tumors phosphatidylcholine (PC) and phosphatidylethanolamine (PE) were reported to be significantly increased [27]. The most significant changes in PC and PE content were observed in the G1 phase of the cell cycle, during which the enzymes that control biosynthesis, catabolism and metabolism of phospholipids attain maximum activity [28,29]. In addition, a high PC/PE ratio was associated with metastases [30]. Li *et al*. also confirmed the relationship of increased PI and PC with colorectal cancer genesis, as well as the relationship between increased PE and hepatic metastasis in colorectal carcinoma [31]. In mammals, phosphatidylserine (PS) plays a role in protein kinase C signaling pathways [32] and is a marker for early apoptosis analysis [33]. Its distribution was altered in different cancers [21]. Sphingosine 1-phosphate (S1P) was originally considered an intracellular second messenger that is involved in the control of cell growth and death signaling pathways [34]. Evidence has proven the critical role of S1P as a tumor-promoting agent [35]. S1P is involved in cancer development through stimulation of cell survival, proliferation, migration, and angiogenesis [36–38]. S1P levels (in a range of 5 μmol/L to 40 μmol/L) were 5 to 10 times upregulated in the ascites of ovarian cancer patient, which stimulates the migration and invasion of epithelial ovarian cancer cells compared with normal ovarian surface epithelial cells. In addition, extracellular S1P have an important role in cancer progression by promoting the migration of epithelial ovarian cancer cells [39]. Therefore, our result suggests that the differentially expressed PL profile may be a potential biomarker for the diagnosis of esophageal cancer because of its statistic and biological significance.

### 2.5. Clinical Estimation of Metabolic Biomarkers with Hierarchical Cluster Analysis

**Figure 3:**
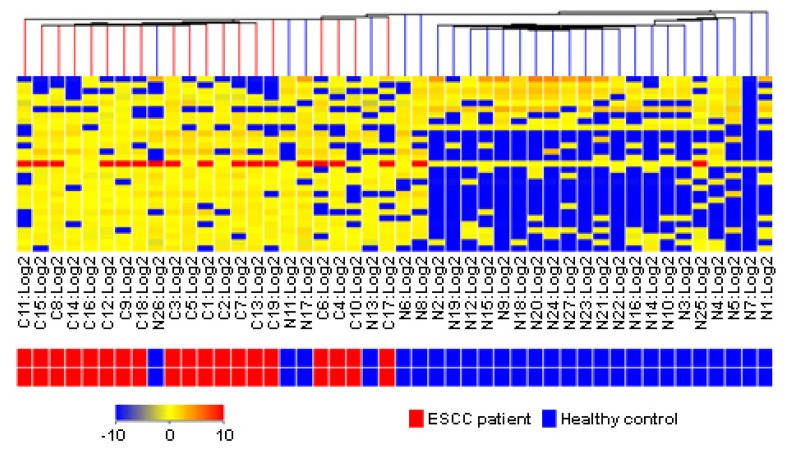
Hierarchical cluster analysis of plasma metabolic profile for distinguishing ESCC patients from healthy controls.

Thirty differential metabolic biomarkers were determined in plasma samples from 17 ESCC patients and 29 healthy volunteers to evaluate the metabolite profile for diagnosing ESCC. Hierarchical cluster analysis was performed based on the metabolite profile. In Figure 3, the rows represent individual samples, and the columns show the results of the expression of metabolite markers. In the bottom bar, the red color indicates ESCC patients, and the blue color indicates healthy individuals. The clustering result indicated that the present metabolite profile could distinguish ESCC patients from healthy individuals.

## 3. Material and Methods

### 3.1. Study Subjects

The present study recruited 53 pairs of ESCC patients and healthy controls from Huaian County of Jiangsu Province, China. Patients were newly diagnosed with histologically confirmed primary cancer and previously untreated (no radiotherapy or chemotherapy) ESCC from October 2008 to December 2009. Healthy control subjects were matched with ESCC patients based on age (±5 years), sex, and residence. The selection criteria included no individual history of cancer and digestive disease. Each subject was scheduled for an interview and a structured questionnaire was administered by the interviewer after informed consent was obtained. Five mL of peripheral blood was collected in heparinized tubes from each subject. Within 6 h after collection, the blood samples were centrifuged by using a three-spin protocol (300× *g* for 30 min, 1200× *g* for 5 min, 2500× *g* for 5 min) to isolate cell-free plasma. Plasma samples were then stored at −80 °C until further processing. The population study was approved by the institutional review board of the Southeast University-affiliated Zhongda Hospital in Nanjing, China.

### 3.2. Sample Preparation and Pretreatment

All plasma samples were thawed in a 4 °C water bath and vortexed for 15 s. A 50 μL aliquot was extracted with 100 μL of methanol and vortexed for 2 min. After being incubated overnight at 4 °C, the mixed solution was centrifuged at 12,000× *g* for 10 min at 4 °C. The supernatant was transferred to another Eppendorf tube for another centrifugation at 12,000× *g* for 10 min at 4 °C. A 20 μL aliquot of supernatant was transferred to a sampling vial pending UPLC-ESI-TOF/MS analysis.

### 3.3. Ultraperformance Liquid Chromatography

A 3 μL aliquot of the pretreated plasma sample was injected into a ZORBAX Eclipse Plus C18 column (3.00 mm × 100 mm, 1.8 μm, Agilent, Santa Clara, CA, USA) by using an ultraperformance liquid chromatography system (Agilent, Santa Clara, CA, USA). Each 5 patient samples were followed by 5 control samples, with an interval of 3 blank samples to avoid cross-contamination. The reference standard was alternately run for each 5 samples for quality control. Then, 0.1% formic acid in water (*v*/*v*) served as mobile phase A, and acetonitrile served as mobile phase B. The gradient elution procedures were as follows: 5% solution B for 0 min to 1 min, 5% to 70% solution B for 1 min to 3 min, 70% to 80% solution B for 3 min to 5 min, 80% to 95% solution B for 5 min to 10 min, 95% solution B for 10 min to 12 min, and 5% solution B for 12 min to 20 min. The flow rate was 0.3 mL/min and column temperature was held at 35 °C. All samples were maintained at 4 °C during the analysis.

### 3.4. Accurate Mass Time-of-Flight Mass Spectrometry

Mass spectrometry was performed by using an accurate mass time-of-flight mass spectrometry 6224 system (Agilent, Santa Clara, CA, USA) equipped with an electrospray ionization source that operates in positive ionization mode (ESI+). The source temperature was set at 110 °C and the desolvation gas temperature was 325 °C with a nebulizing gas flow rate of 9 L/min. Data were collected at a rate of 1 MS spectrum per second from 100 to 1000 *m*/*z* with a scan time of 0.4 s, an inter-scan delay of 0.1 s, and a lock spray frequency of 10 s. The tune mixture solution (Agilent, Santa Clara, CA, USA) was employed as the lock mass (*m*/*z* = 121.050873, 922.009798) at a flow rate of 30 μL/min, via a lock spray interface for accurate mass measurement.

### 3.5. Data Preprocessing and Annotation

MassHunter workstation software (Agilent Technologies, Barcelona, Spain) was used to analyze the accurate mass MS profiling data and extract molecular features. The feature extraction and correlation algorithms located the groups of co-variant ions in each chromatogram. Each of these groups represented a unique compound. After locating the components, the background was subtracted, and the charge state was set to 1. The algorithm identified salt adducts (Na^+^ and K^+^), and the protonated molecules [M + H]^+^ and associated adduct ions were treated as a single compound. The monoisotopic mass and retention time was reported for each feature. An empirical formula was calculated for each feature by using the monoisotopic mass and isotope ratios. Samples were selected with a minimum absolute abundance of 2000 counts and a minimum of 2 ions. Compounds from different samples were aligned by using a RT window of 0.2% ± 0.15 min and a mass window of 10 ppm ± 2.0 mDa, correcting for individual bias.

### 3.6. Statistics

The molecular features extracted by the MassHunter workstation software were aligned and normalized followed by hierarchical clustering to check data quality. ANOVA was performed to identify features with differential abundances across groups. PCA was carried out to select distinct variables as potential biomarkers for distinguishing ESCC patients from healthy controls. All statistical analyses were conducted by using Mass Profiler Professional Software (Agilent Technologies, Barcelona, Spain) at a 5% significance level.

### 3.7. Metabolite Identification

The identification of the candidate biomarkers was based on retention behavior, mass assignment, and online database query [40]. The accurate mass and structure information of candidate metabolites were matched with those of metabolites obtained from HMDB (www.hmdb.ca), METLIN (metlin.scripps.edu/) and KEGG (www.genome.jp/kegg/) databases [41,42]. The mass tolerance between the measured *m*/*z* values and the exact mass of the components of interest was set to within 10 mDa.

## 4. Conclusions

In the present study, a metabolic profiling of plasma including 39 metabolites was constructed for the diagnosis of ESCC by using UPLC-ESI-TOFMS. The proposed protocol determined 25 upregulated molecules and five downregulated molecules. Among 11 molecules identified by databases, six upregulated molecules of interest in ESCC belong to phospholipids as follows: phosphatidylserine, phosphatidic acid, phosphatidyl choline, phosphatidylinositol, phosphatidyl ethanolamine, and sphinganine 1-phosphate. Clinical estimation of metabolic biomarkers with hierarchical cluster analysis in plasma samples from 17 ESCC patients and 29 healthy volunteers indicated that the present metabolite profile could identify ESCC patients from healthy individuals. The cluster of aberrant expression of phospholipids in ESCC indicates that phospholipid metabolism plays a critical role in the oncogenesis of ESCC, which offers insight into the mechanism of carcinogenesis. In addition, a bile acid, lithocholic acid taurine conjugate, was also significantly increased in the plasma of ESCC patients. Downregulated molecules of interest included desmosine/isodesmosine and 5-β-cyprinol sulfate. All the abnormal levels of these metabolites in the plasma of ESCC patients provide new insights into the occurrence and development of the disease.

## Figures and Tables

**Table 1:** Characteristics of ESCC patients and controls.

Variable	No. of patients (*n* = 53)	No. of controls (*n* = 53)
Age (in years)	62.6 ± 7.3	63.2 ± 8.1

Gender		
Male	31	31
Female	22	22

Smoking index a		
Non-user	27 *	40
Ever b		
<400	6	2
≥400	20	11

Alcohol use		
Never	24 *	43
Ever c	29	10

Family history		
No	47 *	53
Yes	6	0

* Significant from control *p* < 0.05 by Student’s *t*-test for age, χ^2^ test for smoking, alcohol use, and family history;

a smoking index = cigarettes/day × smoking years;

b smoked more than 100 cigarettes in lifetime;

c drank at least once a week.

**Table 2:** Plasma differential metabolites associated with ESCC risk.

No.	tR/min	*m*/*z*	Tendency (cancer/control)	Fold change	*p* value	FDR	Postulated identity
1	7.0917454	837.5655	up	1182.88	1.43 × 10^−8^	2.86 × 10^−6^	phosphatidylinositol
2	7.077058	881.5899	up	1046.25	9.10 × 10^−9^	2.73 × 10^−6^	
3	7.9524574	775.527	up	887.53	7.62 × 10^−10^	4.57 × 10^−7^	
4	8.084705	687.471	up	716.17	3.98 × 10^−7^	5.97 × 10^−5^	
5	7.06508	925.6148	up	667.50	4.42 × 10^−7^	5.30 × 10^−5^	
6	7.6628757	777.5422	up	664.51	2.72 × 10^−6^	2.33 × 10^−4^	
7	6.780679	484.3174	up	318.85	2.45 × 10^−5^	1.63 × 10^−3^	Lithocholyltaurine
8	7.091731	842.5196	up	315.36	1.03 × 10^−4^	5.62 × 10^−3^	
9	7.4925284	953.65	up	304.278	1.14 × 10^−5^	8.55 × 10^−4^	
10	6.6252184	487.3345	up	279.64	1.48 × 10^−6^	1.48 × 10^−4^	phosphatidic acid
11	7.2552686	939.634	up	154.73	1.55 × 10^−4^	7.15 × 10^−3^	
12	6.7809725	638.3852	up	146.04	9.14 × 10^−5^	5.48 × 10^−3^	L-Urobilinogen
13	7.108005	798.4944	up	94.10	2.87 × 10^−4^	1.15 × 10^−2^	
14	7.4301453	520.8508	up	92.60	3.04 × 10^−4^	1.14 × 10^−2^	
15	6.925951	784.4782	up	73.87	3.03 × 10^−3^	5.86 × 10^−2^	9′-carboxy-gama-tocotrienol
16	7.108472	793.5397	up	72.40	3.22 × 10^−4^	1.14 × 10^−2^	phosphatidyl choline
17	6.9349127	735.4974	up	65.69	1.88 × 10^−3^	3.88 × 10^−2^	
18	7.4347086	675.474	up	56.34	1.44 × 10^−3^	3.61 × 10^−2^	phosphatidyl ethanolamine
19	7.4338794	395.8942	up	38.66	7.39 × 10^−4^	2.33 × 10^−2^	
20	7.3625846	763.5281	up	27.46	1.72 × 10^−3^	3.69 × 10^−2^	sphinganine 1-phosphate
21	7.124603	749.513	up	26.86	1.40 × 10^−3^	3.66 × 10^−2^	phosphatidylserine (16:0/14:0)
22	7.142187	705.487	up	25.72	9.67 × 10^−3^	1.49 × 10^−1^	
23	7.1617465	661.4607	up	24.96	2.90 × 10^−3^	5.80 × 10^−2^	
24	6.9430337	696.4259	up	22.81	8.45 × 10^−3^	1.33 × 10^−1^	
25	7.1857386	617.434	up	22.67	8.05 × 10^−3^	1.31 × 10^−1^	LysoPC(22:2(13Z,16Z))
26	7.43357	166.9863	up	21.01	5.30 × 10^−3^	9.09 × 10^−2^	
27	6.331661	792.7367	up	19.60	1.54 × 10^−3^	3.69 × 10^−2^	
28	7.1242256	754.4663	up	18.79	4.46 × 10^−3^	7.86 × 10^−2^	Ganglioside GM2 (d18:1/24:1(15Z))
29	6.782452	594.3591	up	18.78	1.19 × 10^−3^	3.39 × 10^−2^	Lithocholate 3-*O*-glucuronide
30	7.144019	710.4422	up	18.49	1.78 × 10^−3^	4.10 × 10^−2^	
31	7.395843	719.5016	up	18.41	3.16 × 10^−3^	5.92 × 10^−2^	
32	6.6147885	443.3086	up	17.67	1.07 × 10^−3^	3.22 × 10^−2^	
33	6.6157866	448.2641	up	12.53	5.60 × 10^−3^	9.34 × 10^−2^	12-oxo-20-dihydroxyleukotriene B4
34	6.485909	434.249	up	8.25	3.93 × 10^−3^	7.14 × 10^−2^	
35	9.1121235	527.3036	down	−13.11	1.19 × 10^−3^	3.25 × 10^−2^	Desmosine/Isodesmosine
36	9.865172	628.3065	down	−25.38	2.19 × 10^−3^	4.86 × 10^−2^	
37	8.604863	445.2468	down	−78.77	2.01 × 10^−4^	8.61 × 10^−3^	
38	7.0976486	533.3094	down	−107.32	4.36 × 10^−4^	1.45 × 10^−2^	5-β-cyprinol sulfate
39	8.611813	586.2785	down	−233.30	1.11 × 10^−4^	5.55 × 10^−3^	
